# Dynamic Analysis of Photosynthate Translocation Into Strawberry Fruits Using Non-invasive ^11^C-Labeling Supported With Conventional Destructive Measurements Using ^13^C-Labeling

**DOI:** 10.3389/fpls.2018.01946

**Published:** 2019-01-09

**Authors:** Kota Hidaka, Yuta Miyoshi, Satomi Ishii, Nobuo Suzui, Yong-Gen Yin, Keisuke Kurita, Koyo Nagao, Takuya Araki, Daisuke Yasutake, Masaharu Kitano, Naoki Kawachi

**Affiliations:** ^1^Kyushu Okinawa Agricultural Research Center, National Agriculture and Food Research Organization, Kurume, Japan; ^2^Takasaki Advanced Radiation Research Institute, National Institutes for Quantum and Radiological Science and Technology, Takasaki, Japan; ^3^Postdoctoral Research Fellow of the Japan Society for the Promotion of Science, Tokyo, Japan; ^4^Faculty of Agriculture, Kyushu University, Fukuoka, Japan; ^5^Faculty of Agriculture, Ehime University, Matsuyama, Japan

**Keywords:** fruit position, PETIS, photosynthate, strawberry, translocation, visualization

## Abstract

In protected strawberry (*Fragaria* × *ananassa* Duch.) cultivation, environmental control based on the process of photosynthate translocation is essential for optimizing fruit quality and yield, because the process of photosynthate translocation directly affects dry matter partitioning. We visualized photosynthate translocation to strawberry fruits non-invasively with ^11^CO_2_ and a positron-emitting tracer imaging system (PETIS). We used PETIS to evaluate real-time dynamics of ^11^C-labeled photosynthate translocation from a ^11^CO_2_-fed leaf, which was immediately below the inflorescence, to individual fruits on an inflorescence in intact plant. Serial photosynthate translocation images and animations obtained by PETIS verified that the ^11^C-photosynthates from the source leaf reached the sink fruit within 1 h but did not accumulate homogeneously within a fruit. The quantity of photosynthate translocation as represented by ^11^C radioactivity varied among individual fruits and their positions on the inflorescence. Photosynthate translocation rates to secondary fruit were faster than those to primary or tertiary fruits, even though the translocation pathway from leaf to fruit was the longest for the secondary fruit. Moreover, the secondary fruit was 25% smaller than the primary fruit. Sink activity (^11^C radioactivity/dry weight [DW]) of the secondary fruit was higher than those of the primary and tertiary fruits. These relative differences in sink activity levels among the three fruit positions were also confirmed by ^13^C tracer measurement. Photosynthate translocation rates in the pedicels might be dependent on the sink strength of the adjoining fruits. The present study established ^11^C-photosynthate arrival times to the sink fruits and demonstrated that the translocated material does not uniformly accumulate within a fruit. The actual quantities of translocated photosynthates from a specific leaf differed among individual fruits on the same inflorescence. To the best of our knowledge, this is the first reported observation of real-time translocation to individual fruits in an intact strawberry plant using ^11^C-radioactive- and ^13^C-stable-isotope analyses.

## Introduction

Strawberry fruits have sweet and tart tastes, pleasant flavor, and antioxidant properties. Their exceptional palatability and health benefits make them very important in the global fruit crop market (Hancock, 1999). Strawberry production in protected facilities (greenhouses) has been globally increasing to enhance yield and quality of fruits by avoiding the adverse environments, and to extend harvest period to near year-round by the improvement of temperature environment (Neri et al., 2012; Garcia et al., 2017). In protected strawberry cultivation, an introduction of environmental control techniques, such as CO_2_ enrichment, supplemental lighting has increased to enhance photosynthesis and improve fruit yield and quality (Sung and Chen, 1991; Deng and Woodward, 1998; Hidaka et al., 2013, 2016; Miyoshi et al., 2017). This requires optimizing both photosynthesis and photosynthate translocation into developing fruit.

Photosynthate translocation from the leaves (sources) to the harvestable target organs (sinks) directly affects dry matter accumulation there and promotes auxetic growth of the sink tissues, and significantly influences crop yield and quality (Troughton and Currie, 1977; Braun et al., 2014). In the production of fruit crops like tomato, cucumber, and strawberry, effective photosynthate translocation to the sink fruit is extremely important since these are the harvestable organs. Dry matter partitioning to the sink is primarily regulated by the sink itself, and the ability of the target organ to attract assimilates is called sink strength (Gifford and Evans, 1981; Marcelis, 1996). Sink strength is determined by sink size and sink activity. The latter reflects physiological responses in translocation (Ho, 1996). In translocation, photosynthate is loaded from the source organs to the phloem then unloaded from the phloem to the sink organs. This process is largely influenced by environmental factors like air temperature, light intensity, and CO_2_ concentration (Lemoine et al., 2013). Thus, to obtain high fruit yield and quality in strawberry plants, it is essential to clarify the sink-source relationship and translocation responses to environmental conditions, and to regulate the sink-source balance (numbers of leaves and fruits) and the cultivation environment based on its obtained knowledge for optimization of photosynthate translocation into the sink fruits. In tomato plants, the sink-source relationship and translocation responses to the environmental conditions have been investigated and established (Ho, 1988; Kitano et al., 1998; Shishido et al., 1999; Araki et al., 2001; Heuvelink, 2005). Shishido and Hori (1977) reported that in tomato plants, photosynthate distribution to the fruiting inflorescence from the leaves immediately below it was remarkably higher than that from the leaves immediately above it. However, such studies have not yet been adequately conducted in strawberry plants. The strawberry plant stem is a short, thick structure known as the crown. It is located at the base of the plant body. The photosynthate transport pathway between each petiole (source leaf side) and peduncle (sink fruit side) is connected at the crown. The short strawberry stem has a dense vascular system relative to tomato plant stem. The anatomy of strawberry makes it histophysiologically difficult to clarify its sink-source relationship. Forney and Breen (1985a) analyzed phloem exudate from strawberry pedicels by the EDTA method. The influences of growth stage (vegetative, reproductive), inflorescence development, leaf position, and environmental conditions (light intensity, air temperature) on photosynthate translocation in strawberry were examined by the ^14^C and ^13^C tracer methods (Nishizawa and Hori, 1986, 1988; Kumakura and Shishido, 1994; Nishizawa et al., 1998; Hidaka et al., 2014). However, translocation measurements determined by the aforementioned techniques require the destruction and extraction of plant tissues. Therefore, the data obtained for a given analytical period are fragmentary and must be integrated. Furthermore, previous studies implementing these techniques assessed translocation to entire inflorescences rather than individual fruits. Nevertheless, individual commercial strawberry fruit quality (sweetness, sourness, flavor) is evaluated and must be consistently high. According to Watson et al. (2002), the strawberry fruit flavor is largely influenced by the quantity of translocated photosynthate, because the latter is a precursor of the volatile organic compounds responsible for fruit flavor. Therefore, adequate photosynthate translocation to each individual fruit on an inflorescence is necessary for the production of a stable high-quality fruits in strawberry plants. In practice, however, translocation to individual fruits may vary even on the same inflorescence. It is widely accepted that the phloem transport of sucrose, one of main translocational sugars in strawberry (Forney and Breen, 1985a), is driven by mass-flow (Münch, 1930) and by hydrostatic pressure differences in the phloem between source and sink. Relatively higher phloem sucrose concentrations upstream (source side) than downstream (sink side) are maintained by active phloem loading via energy-consuming proton/sucrose co-transport mediated by sucrose transporters (SUTs) and H^+^/ATPase. Chiou and Bush (1998) hypothesized that phloem sucrose loading is regulated by the alterations in SUT activity resulting from transductional sucrose signaling in response to changes in sink strength. Bangerth (1989) proposed that photosynthate partitioning among competing sink fruits is regulated by auxin as the dominance signals from sink fruits. Therefore, photosynthate partitioning among competing sink fruits on the same inflorescence could be determined by sink strength and/or sink dominance of each fruit (Lemoine et al., 2013). In these processes, sucrose and auxin signals may play key roles. To date, spatiotemporal-continuous translocation to individual strawberry fruits on the same inflorescence in intact plants has never been evaluated, and this investigation could contribute to elucidate the photosynthates translocation mechanism in strawberry plants.

Positron-emitting tracer imaging system (PETIS) can non-invasively visualize the dynamics of spatiotemporal-continuous translocation of photosynthates with ^11^C-photosynthate detection (Kawachi et al., 2006). Translocation data obtained with this system reflects real-time photosynthate transport in the phloem whereas ^13^C-tracer data reflects the total process including translocation, retranslocation to the other organs, and respiratory photosynthate consumption. We have already successfully evaluated real-time photosynthate translocation in tomato and eggplant using PETIS (Kikuchi et al., 2008; Yamazaki et al., 2015). In this study, dynamics of real-time photosynthate translocation from an individual leaf immediately below the inflorescence, which is the leaf position observed the high photosynthate partitioning to the inflorescence in tomato plants as aforementioned (Shishido and Hori, 1977), to intact strawberry fruits was examined by using PETIS for the first time. It was also used to compare relative photosynthate translocation rates to individual fruits on the same inflorescence, and further the results were confirmed by the ^13^C-tracer method to support the results of ^11^C data.

## Materials and Methods

### Plant Material and Growth Conditions

June bearing strawberries (*Fragaria* × *ananassa* Duch. cv. Fukuoka S6) were grown in a 37 m long × 9 m wide section of a greenhouse (37 m long × 27 m wide × 4.5 m high) at the NARO Kyushu Okinawa Agricultural Research Center, Japan (33°18.4′N, 130°32.8′E). In early June, nursery plants were selected from mother stocks and transplanted into plastic pots (6 cm diameter; 0.2 L volume). Connections to the mother stocks were retained through runners. Pots were filled with a substrate consisting of peat moss, coconut shells, and charcoal (3:5:2 [v/v/v]) and placed on a nursery bench. The plants were irrigated with plain water until rooting when their runners were cut from the mother stocks (late June). Thereafter, they were supplied with a nutrient solution (OK-F-1, OAT Agrio Co., Ltd., Tokyo, Japan; electrical conductivity = 0.6 dS m^−1^) at a rate of 300 mL d^−1^/plant. Nutrient supplementation was suspended from mid-August to mid-September to induce anthesis. During this time, only water was supplied. By mid-September, flower buds had differentiated on the first inflorescences. The nursery plants were transplanted into substrate-filled plastic pots (0.15 m diameter; 2.6 L volume) and set on cultivation beds (30 m long × 30 cm wide × 80 cm high) at intervals of 20 cm between plants and 15 cm between rows. Henceforth, they were supplied with nutrient solution. The substrates and nutrient solutions were the same as those described above. For air temperature control in the greenhouse, the threshold temperature of the heater (HK2027TEV, NEPON Inc., Tokyo, Japan) was set to 8°C in the nighttime, and the daytime ventilation temperature was set to 27°C. Flowers were pollinated by bees. After anthesis, the plants were analyzed by PETIS and ^13^C tracer method explained later.

### Positron-Emitting Tracer Imaging System (PETIS)

PETIS is a non-invasive, real-time imaging technique for the analysis of plant physiological functions. It uses radioisotope tracers to produce movie-like serial two-dimensional images of long-distance translocation in plant bodies. PETIS imaging is based on the coincident detection of γ-rays emitted in opposite directions at 511 keV generated by the annihilation of an electron (e^−^) and a positron (β^+^) emitted from a radionuclide-labeled tracer. The principle of PETIS-based image acquisition is described in Uchida et al. (2004).

**Figure 1:**
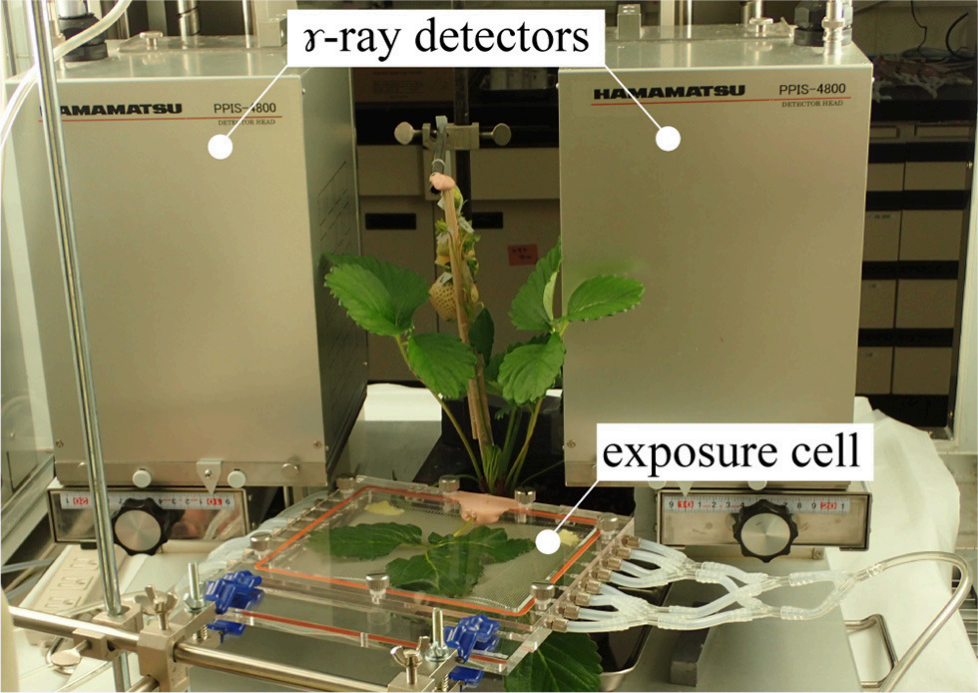
Photograph of the PETIS experimental setup. A test plant was placed between the γ-ray detectors in the PETIS field of view. ^11^CO_2_ was fed to the leaf through an exposure cell. Images of ^11^C labeled photosynthate translocation were continuously acquired every 10 s. PETIS was installed in a plant growth chamber. Photosynthetically active radiation (*PAR*), air temperature (*TA*), relative humidity (*RH*), and CO_2_ concentration could be controlled inside the growth chamber according to the experimental setup.

Figure 1 shows the setup of PETIS experiment. PETIS was installed in a plant growth chamber so that the ambient environmental conditions (photosynthetically active radiation (*PAR*), air temperature (*TA*), relative humidity (*RH*), and CO_2_ concentration) could be completely controlled during the experiment. The apparatus was a modification of a PPIS-4800 (Hamamatsu Photonics, Hamamatsu, Japan) and consisted of two plane detector heads fitted with positron-sensitive γ-ray scintillation detectors using a Bi_4_Ge_3_O_12_ (BGO) crystal detector module (Nagai et al., 1996). The detector heads were facing each other and separated by 20 cm. To evaluate photosynthate translocation dynamics, a test plant was placed in a transparent acrylic box (exposure cell) which was then set between the two detectors. Then ^11^C-labeled carbon dioxide (^11^CO_2_) gas was injected into the exposure cell. The ^11^C assimilated by the test plant body emitted two annihilation γ-rays in opposite directions. The two PETIS detectors simultaneously captured these annihilation γ-rays (within 20 ns) and identified the γ-ray emission point from the test plant as the point midway between the two detection points. These events were recorded on a personal computer and converted to focal-plane coordinate data to produce a two-dimensional image. The images were acquired every 10 s by histogram memory in the personal computer. The typical field of view was 12 cm wide × 19 cm high. The spatial resolution was ~0.2 cm. Therefore, it is difficult for imaging the detailed distribution and translocation within a fruit, but it is suitable for imaging of translocation between plant organs. We carried out an experiment for a semiannual maintenance of the sensitivity correction of the PETIS detectors using a flat uniform phantom filled with radioactive solution.

### ^11^CO_2_ Tracer Production

The ^11^CO_2_ was produced from ^14^N(p, α) ^11^C by bombarding N_2_ with an energetic proton beam generated by an AVF cyclotron at Takasaki Ion Accelerators for Advanced Radiation Application (TIARA), National Institutes for Quantum and Radiological Science and Technology (QST), Japan (Ishioka et al., 1999; Yamazaki et al., 2015). Approximately 200 MBq ^11^CO_2_ was collected in a stainless-steel trap immersed in liquid nitrogen and used for PETIS measurement.

### PETIS Measurement

Strawberry plants at the third inflorescence fruiting stage were brought into the TIARA and cultivated in a plant growth chamber at 12 h photoperiod, *PAR* = 500 μmol m^−2^ s^−1^, *TA* = 20°C, *RH* = 60%, and CO_2_ concentration = 380 μmol mol^−1^. Nutrient solution was supplied as described above at 300 mL d^−1^ plant^−1^. The source and sink consisted of nine leaves and six fruits, respectively.

**Figure 2:**
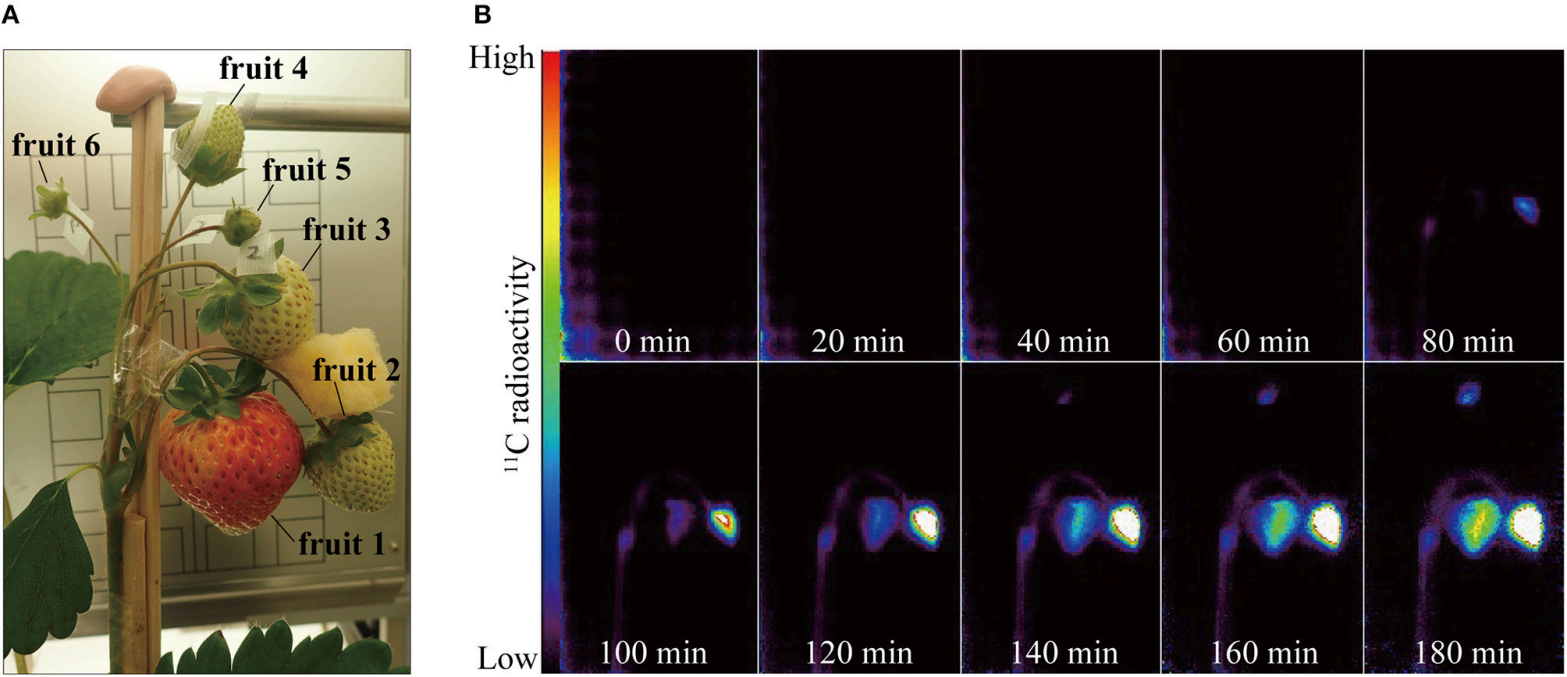
**(A)** Photograph of strawberry fruits in the PETIS field of view. Positions of the respective fruits on the same inflorescence were as follows: fruit 1, primary; fruits 2 and 3, secondary; fruits 4, 5, and 6, tertiary. **(B)** Serial PETIS images of ^11^C-photosynthate translocation to the fruits. Each image in **(B)** was an integration of 120 serial images continuously acquired by PETIS measurement every 10 s for 180 min.

Strawberry plants were placed between two opposing PETIS detectors. The fruits were fixed with cellophane tape in the field of view of the PETIS so that they were correctly oriented on the focal plane and did not mutually intercept their fields of view. Figure 2A shows the strawberry fruits in the PETIS field of view. The six fruits on the three different positions within an inflorescence were categorized into the following positions and developmental stages: fruit 1 (primary fruit; turning red stage); fruits 2 and 3 (secondary fruit; white stage); fruits 4, 5, and 6 (tertiary fruit; green stage). A fourth leaf developing immediately below the inflorescence was inserted into the exposure cell (20 cm in length, 15 cm in width, and 1 cm in depth; inside dimensions) which was then sealed with plastic clay at the petiole entry point to prevent ^11^CO_2_ leakage. The exposure cell was then connected to a ^11^CO_2_ gas circulation system consisting of pumps, mass flow controllers, electric valves, and a CO_2_ trap containing freshly prepared soda lime. At the start of imaging, room air was fed into the exposure cell. Then 200 MBq ^11^CO_2_ mixed with room air was introduced from the CO_2_ trap into the exposure cell for 20 min. The ^11^CO_2_ was then flushed off and substituted with non-radioactive room air. PETIS images were acquired every 10 s for 180 min. The image data were automatically calibrated for the ^11^C decay assuming a half-life of 20.39 min and recorded on a personal computer. The environment in the growth chamber around the PETIS was controlled at *PAR* = 500 μmol of photons m^−2^ s^−1^, *TA* = 20°C, *RH* = 60%, and CO_2_ concentration = 380 μmol mol^−1^.

### Non-invasive Translocation Analysis

**Figure 3:**
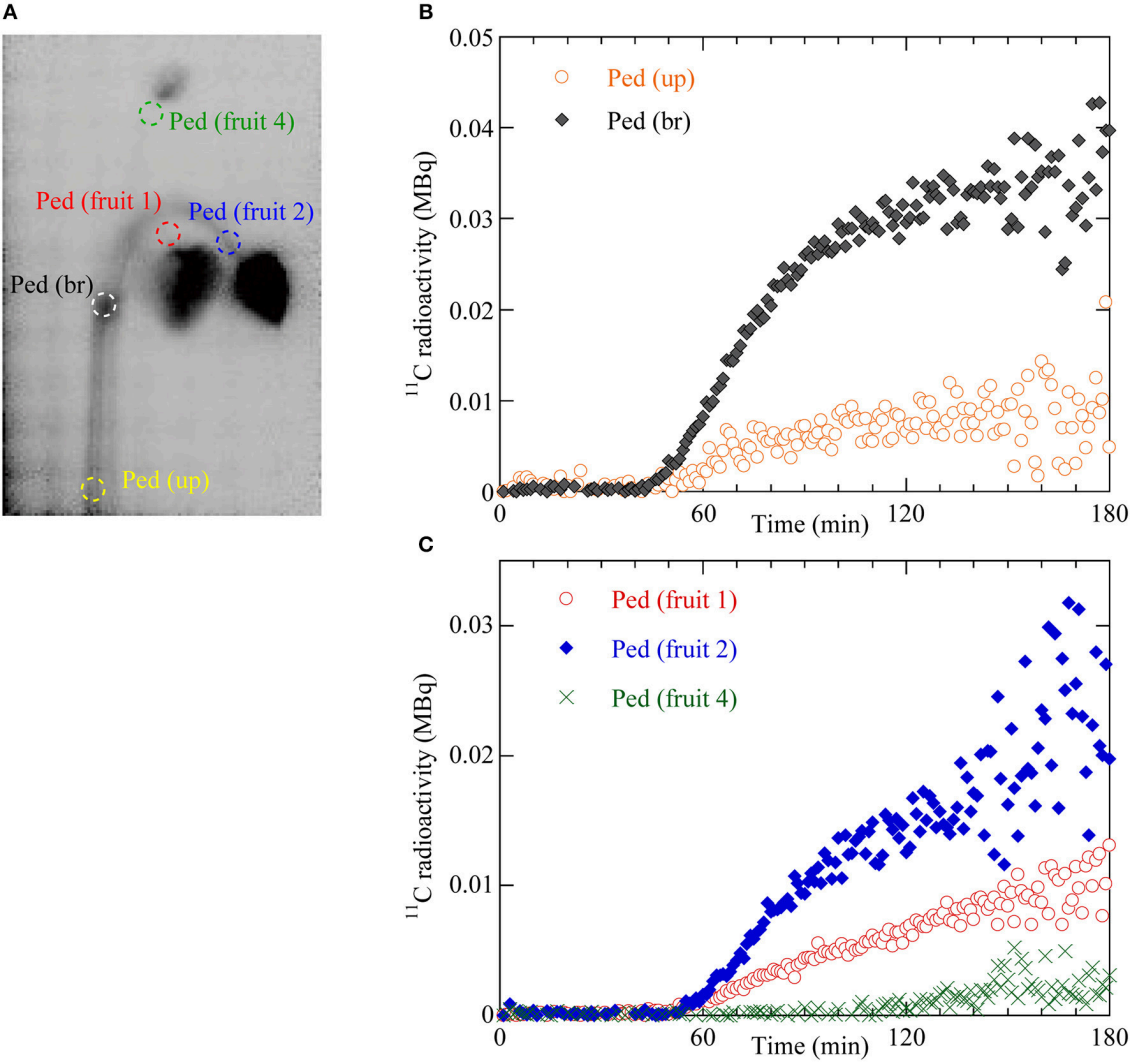
**(A)** Regions of interest (ROIs) on the integrated PETIS image. Red dotted ellipse indicates the ROI of the pedicel of fruit 1 (Ped fruit 1). Blue dotted ellipse indicates the ROI of the pedicel of fruit 2 (Ped fruit 2). Green dotted ellipse indicates the ROI of the pedicel of fruit 4 (Ped fruit 4). White dotted ellipse indicates the ROI of the branching peduncle (Ped br). Yellow dotted ellipse indicates the ROI of the upstream part of the peduncle (Ped up). **(B)** Time courses of ^11^C radioactivity in Ped (br) and Ped (up). **(C)** Time courses of ^11^C radioactivity in Ped (fruit 1), Ped (fruit 2), and Ped (fruit 4).

**Figure 4:**
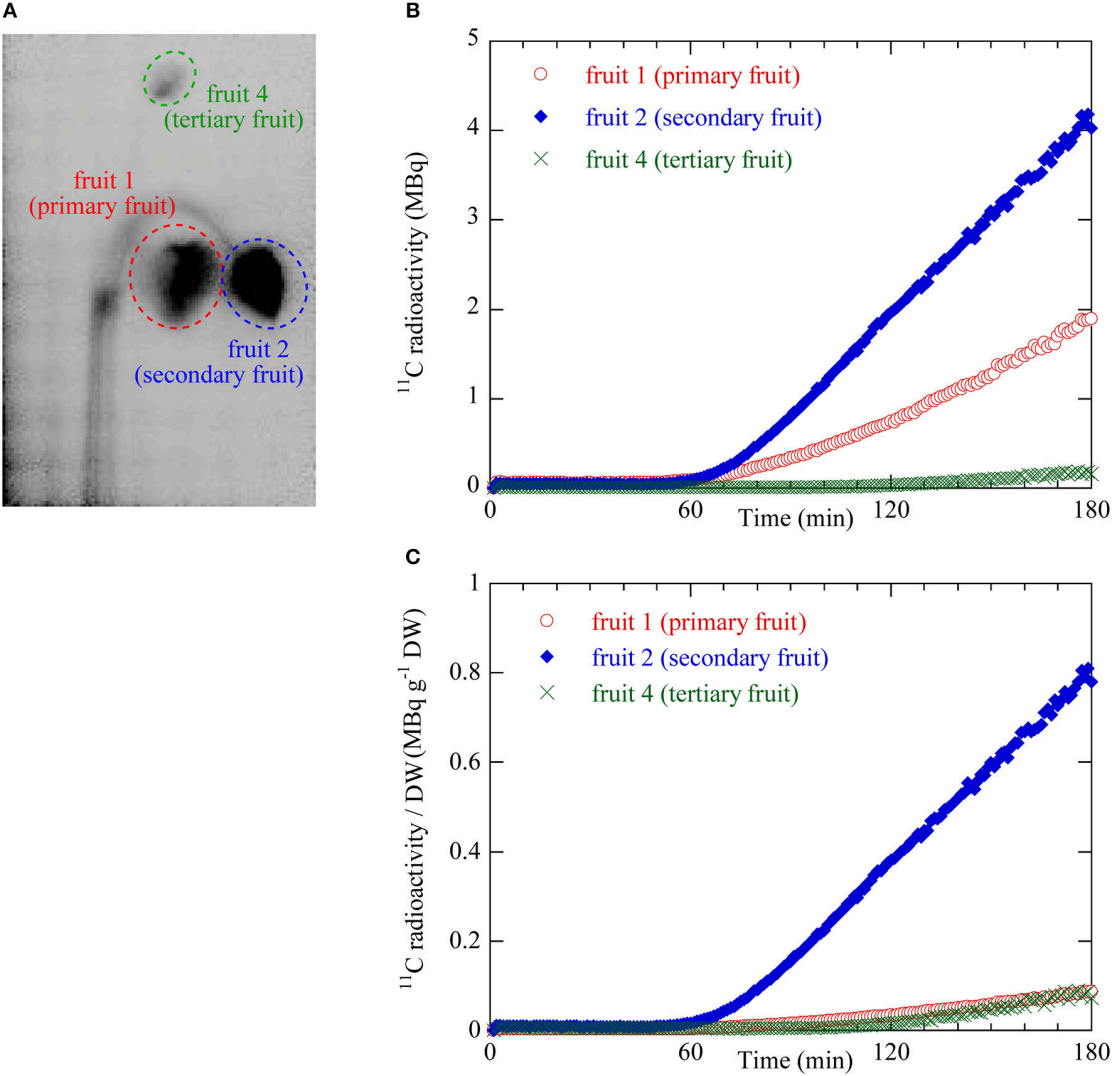
**(A)** ROIs on the integrated PETIS image. Red dotted ellipse indicates the ROI for fruit 1 (primary) shown in Figure 2A. Blue dotted ellipse indicates the ROI of fruit 2 (secondary). Green dotted ellipse indicates the ROI of fruit 4 (tertiary). ^11^C radioactivity measurement inside the ROIs generated time courses of ^11^C radioactivity at the various fruit positions. **(B)** Time courses of ^11^C radioactivity in fruit 1 (primary), fruit 2 (secondary), and fruit 4 (tertiary). **(C)** Time courses of ^11^C radioactivity per fruit dry weight (DW). These values were taken as the sink activity levels in fruit 1 (primary), fruit 2 (secondary), and fruit 4 (tertiary).

The dynamics of ^11^C translocation through the peduncles into the fruits were analyzed by defining regions of interest (ROIs) around the peduncles, pedicels, and fruits on the PETIS images (Figures 3A, 4A). Because there is uniform sensitivity to positron-emitting radionuclide within the fields of view of PETIS, ^11^C radioactivity of different fruits can be quantitatively compared. Time courses of ^11^C radioactivity (Bq) within each ROI were generated from the signal intensities (cps) obtained with Image J v. 1.50 (National Institutes of Health, Bethesda, MD, USA; http://rsb.info.nih.gov/ij/). The counting efficiency of the system (cps Bq^−1^) was then calculated. In this study, ^11^C radioactivity per fruit (Figure 4B) was taken to represent real-time photosynthate accumulation in individual fruits, and the ^11^C radioactivity per fruit per unit dry weight (DW) was defined as the fruit sink activity as described previously (Hori and Shishido, 1977).

### ^13^C Tracer Measurement

The effects of fruit position on photosynthate translocation from leaves to fruits were evaluated by partitioning ^13^C-photosynthates to the different fruit positions on an inflorescence. The carbon translocation analysis was conducted in a growth chamber in which the photoperiod, *PAR, TA, RH* and CO_2_ concentration were controlled as they were for the PETIS measurement. Plant materials consisted of the remaining three plants not used for PETIS measurement. Leaves and fruits were not thinned over the course of the experiment and the plants remained intact. The source and sink consisted of 7–10 leaves and 4–5 fruits, respectively. Within an inflorescence, there were one primary fruit, two secondary fruits, and one or two tertiary fruits which were the red, white, and green stages, respectively. In ^13^C-tracer measurement, an individual leaf immediately below the inflorescence was fed ^13^C-labeled CO_2_ gas, which was also used in ^11^C-labeled PETIS measurement. During ^13^C feeding, the assimilation leaf immediately below the inflorescence was enclosed in a vinyl film bag (40 cm long × 28 cm wide) containing a 50-mL test tube with 0.5 g Ba^13^CO_3_. Then, 10 mL of 10% (v/v) lactic acid was injected into the test tube with a disposable syringe. During the ^13^CO_2_ feeding, *PAR* on the leaf surfaces was set to 500 μmol photons m^−2^ s^−1^. After 2 h exposure to ^13^CO_2_, the vinyl bag was removed. Three plants were sampled for measurement. At 24 h after ^13^CO_2_ feeding, the plants were collected and separated into leaf, crown, peduncle, fruit, and root. Each part was dried for 48 h at 80°C and pulverized in a vibration mill. The ^13^C content was measured with a gas chromatography combustion isotope ratio mass spectrometer (Integra CN, Sercon Ltd., Crewe, UK). Fruit sink activity was calculated by dividing the fruit ^13^C content by the fruit DW as described in the ^11^C translocation analysis.

### Statistical Analysis

For the ^13^C photosynthate translocation data, mean values of ^13^C-photosynthate content in fruits were based on three replicate plants (*n* = 3). The significance of the differences between means (*P* < 0.05) among the fruit positions on an inflorescence was determined by the Tukey-Kramer test. Statistical analyses were performed in the SAS add-in for Microsoft Office v. 7.15 (SAS Institute Inc., Cary, NC, USA).

## Results

### Non-invasive Visualization and Dynamic Analysis of Photosynthate Translocation

Figure 2B shows serial PETIS images of ^11^C translocation taken at 20-min intervals after ^11^CO_2_ feeding. These were integrations of 120 serial images acquired every 10 s. No ^11^C-photosynthate was detected in the fruits in the PETIS field of view for the first 40 min after the ^11^CO_2_ feeding. About 80 min after the ^11^CO_2_ feeding, ^11^C-photosynthate translocation was visually confirmed via the peduncle to fruits 1 and 2. After another 60 min, ^11^C-photosynthate reached fruit 4. Photosynthate accumulation in the fruits was not homogeneous. In which it, photosynthate accumulation started on the right side of fruit 1 and the upper part of fruit 2. Thereafter, photosynthate accumulation in fruits 1, 2, and 4 gradually increased over time. At 180 min after ^11^CO_2_ feeding, the quantities of accumulated photosynthates were relatively higher on the right side of fruit 1 and the lower part of fruit 4. Nevertheless, photosynthate accumulated everywhere in fruit 2. The ^11^C-photosynthate was not translocated to fruits 3, 5, or 6.

To quantify the dynamics of ^11^C-labeled photosynthate translocation from the leaf to the fruits, we drew circular regions of interest (ROIs) on the PETIS image on the upstream (Ped up) and branching (Ped br) parts of the peduncle, the pedicels of fruits 1, 2, and 4 (Figure 3A), and around fruits 1, 2, and 4 (Figure 4A). Figure 3B shows ^11^C radioactivity time courses (time-activity curves; TACs) in each ROI of Ped (up) and Ped (br) after ^11^CO_2_ feeding. TACs were generated from serial PETIS images acquired every 10 s over 180 min of PETIS measurements. TACs were corrected for ^11^C decay. The ^11^C radioactivity levels in Ped (up) and Ped (br) began to increase at 43 and 44 min after ^11^CO_2_ feeding, respectively. The ^11^C radioactivity levels in Ped (up) and Ped (br) reached plateaus of 0.01 and 0.03 MBq after 120 min. The value for Ped (br) was 3 times greater than that for Ped (up) at 180 min. Figure 3C shows the ^11^C radioactivity time courses in each ROI of Ped (fruit 1), Ped (fruit 2), and Ped (fruit 4). The ^11^C radioactivity levels in Ped (fruit 1), Ped (fruit 2), and Ped (fruit 4) began to increase at 49, 50, and 105 min after ^11^CO_2_ feeding, respectively. The terminal ^11^C radioactivity level in Ped (fruit 2) was 1.5 times and 6.6 times higher than those for Ped (fruit 1) and Ped (fruit 4), respectively.

Figure 4B shows the ^11^C radioactivity time courses after ^11^CO_2_ feeding in each ROI of fruits 1, 2, and 4 (Figure 4A). The ^11^C radioactivity levels in fruits 1 and 2 began to increase 52 min and 55 min after ^11^CO_2_ feeding, respectively. The ^11^C radioactivity level in fruit 4 began to increase 106 min after ^11^CO_2_ feeding. The rate of increase in radioactivity was highest for fruit 2 followed by fruits 1 and 4. The terminal ^11^C radioactivity level in fruit 2 was 2 times and 20 times higher than those in fruits 1 and 4, respectively.

Figure 4C shows the ^11^C radioactivity time courses per DW in each fruit ROI (Figure 4A). The DW of fruits 1, 2, and 4 were 2.19, 0.52, and 0.23 g, respectively. The ^11^C radioactivity level per DW in the ROI of fruit 2 was larger than those in fruits 1 and 4. The terminal ^11^C radioactivity level per DW in fruit 2 was 8 times greater than those of fruits 1 and 4. The terminal ^11^C radioactivity level per DW were very similar for fruits 1 and 4.

### ^13^C-photosynthate Translocation at the Different Fruit Positions

**Figure 5:**
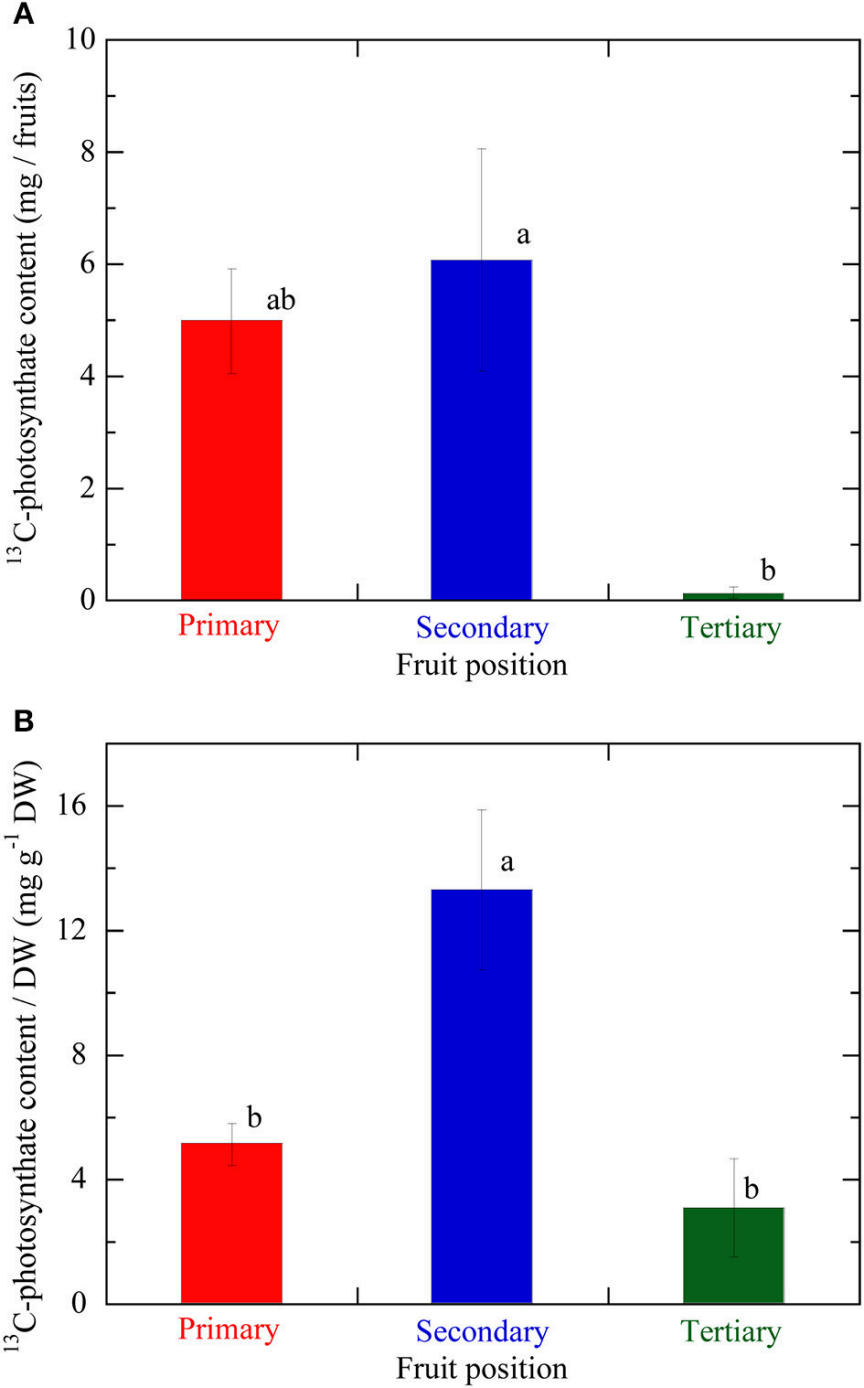
**(A)** ^13^C-photosynthate content in fruits at the different positions on the same inflorescence. **(B)** ^13^C-photosynthate content per fruit DW, which value was taken as the fruit sink activity levels, at the different fruit positions on the same inflorescence. Primary, secondary, and tertiary fruit positions are indicated in red, blue, and green, respectively. Data are mean ± S.E. (*n* = 3). Different letters indicate significant differences among the different fruit positions by Tukey-Kramer test (*P* < 0.05).

Figure 5A illustrates the ^13^C-photosynthate content in fruits at different positions on an inflorescence. The average ^13^C-photosynthate content in the secondary fruit was the highest at all three positions and significantly higher than that of the tertiary fruit. However, no significant difference was detected between the primary and secondary fruit in terms of the ^13^C-photosynthate content. Figure 5B shows the ^13^C-photosynthate content per DW at the various fruit positions on an inflorescence. The average ^13^C-photosynthate content per DW in the secondary fruit was significantly higher than those in the primary and tertiary fruits. The ^13^C-photosynthate content in the primary fruit was higher than that in the tertiary fruit but the difference was not significant.

## Discussion

Several studies have analyzed photosynthate translocation in strawberry. Nishizawa and Hori (1986) examined the influence of inflorescence development on photosynthate translocation by the ^14^C tracer method. Hidaka et al. (2014) evaluated the effects of supplemental LED lighting on photosynthesis induction and augmentation and photosynthate translocation by the ^13^C tracer method. Forney and Breen (1985a) used the EDTA method to identify the major organic compounds in the photosynthate translocated to strawberry fruits. However, the analytical techniques used in these studies were invasive, destructive, and obtained only integrative data over a certain time period. To the best of our knowledge, the present study is the first to analyze photosynthate translocation dynamics in strawberry in a non-invasive, real-time manner using PETIS. The accumulation of ^11^C-labeled photosynthate in strawberry fruits was successful visualized by continuous spatiotemporal animation (Movie S1). It was established from these animations that one source leaf has vascular systems connecting to multiple fruits on an inflorescence and supplies photosynthate to them all. However, the analysis also revealed that photosynthate accumulation was not uniform within a fruit. Heterogeneous photosynthate accumulation within a strawberry fruit has never been previously reported. Shishido et al. (1988) suggested that the configurations of the vascular systems between source and sink affect photosynthate distribution in tomato plants. Kikuchi et al. (2008) reported non-uniform photosynthate accumulation within an eggplant fruit and attributed it to the arrangement of the vascular systems between source and sink. Therefore, the vascular system configuration between source leaf and sink fruit in strawberry may also account for the irregularities in photosynthate accumulation. Quantitative analysis was conducted by setting ROIs on the serial images of ^11^C-photosynthate translocation dynamics. The arrival times of the photosynthate in the fruits were evaluated using the initial increases in the TAC peaks for the photosynthates translocated to the peduncles, pedicels, and fruits in the translocation pathway. The ^11^C-photosynthates assimilated by the individual leaf immediately below the inflorescence were translocated upstream of the peduncle by 43 min after carbon fixation and reached the branching peduncle by 44 min. Thence, the photosynthates were translocated through the pedicels. They arrived at Ped fruits 1, 2, and 4 by 49, 50, and 105 min after photosynthesis, respectively. Finally, they reached fruits 1, 2, and 4 by 52, 55, and 106 min after carbon fixation, respectively. These results suggest that the photosynthates were translocated from the leaf to the peduncle in about 40 min and arrived in the fruit 10 min later.

Analysis of photosynthate translocation to individual strawberry fruits confirmed that photosynthate translocation from a specific leaf was not uniform and varied with fruit position on the same inflorescence. The ^11^C-photosynthate assimilated by the source leaf was translocated to fruits 1, 2, and 4 after 52, 55, and 106 min, respectively. The lengths of the translocation pathways between the petiole and the pedicels differed for each fruit on the inflorescence (Table 1). For fruits 1, 2, and 4, the distances were 34.8, 37.6, and 37.2 cm, respectively. Although the distance between the source leaf and fruit 2 was the longest, the ^11^C-photosynthate translocation rate of fruit 2 was the fastest of all (Figure 4B). It has been reported that photosynthate transport distance, which indicates translocation pathway resistance, does not regulate photosynthate partitioning (Gent, 1982; Heuvelink, 1995). Dry matter partitioning to the fruit is determined mainly by sink strength (Gifford and Evans, 1981; Marcelis, 1996). In the present study, transport distance was not also a primary factor regulating photosynthate translocation rate. Therefore, the differences in the quantity and rates of photosynthate translocation to the fruits might be determined by the relative differences in sink strength among them. TAC analysis of the translocation pathway was used to evaluate the ^11^C-photosynthate translocation rate (Table 1). The photosynthate translocation rates for Leaf-Ped (fruit 1), Leaf-Ped (fruit 2), and Leaf-Ped (fruit 4) were 0.71, 0.75, and 0.35 cm min^−1^, respectively. The photosynthate translocation rates for Ped (br)-Ped (fruit 1), Ped (br)-Ped (fruit 2), and Ped (br)-Ped (fruit 4) were 0.36, 0.77, and 0.07 cm min^−1^, respectively. The photosynthate translocation rates in the Leaf-Ped and Ped (br)-Ped of fruit 2 were almost the same. For fruits 1 and 4, however, the photosynthate translocation rates for Ped (br)-Ped (fruit 1) and Ped (br)-Ped (fruit 4) were lower than those for Leaf-Ped (fruit 1) and Leaf-Ped (fruit 4). Therefore, the photosynthate translocation rates in fruits 1 and 4 might differ at the level of the upstream- and downstream sides of the branching peduncle. On the downstream side of the branching peduncle, the photosynthate translocation rate within the pedicel of fruit 2 was higher than those in fruits 1 and 4. This discrepancy might be explained by the relative differences in sink strength among the fruits. For this reason, the comparative quantities of accumulated photosynthates differed among the fruits as shown in Figure 4. According to Warren (1972), sink strength is the product of sink size and sink activity. However, certain researchers claim that sink size is not an important determinant of sink strength (Moorby, 1968; Marcelis, 1996). In the present study, the photosynthate translocation rate of fruit 2 was greater than that of fruit 1 even though fruit 2 was only about 25% the size of fruit 1 (dry weights 0.52 and 2.19 g, and volumes 56 and 237 cm^3^ for fruit 2 and 1, respectively). Real-time photosynthate translocation analysis in the present study corroborated the hypothesis that sink size does not significantly influence sink strength. Ho (1996) suggested that sink activity reflects the physiological responses of sink organs in translocation. Based on the ^11^C translocational data from a specific leaf to fruits, we evaluated sink activity by normalizing ^11^C radioactivity to fruit DW. The sink activity of fruit 2 (secondary fruit) was higher than those for the other fruits. The sink activity levels of fruit 1 (primary fruit) and fruit 4 (tertiary fruit) were nearly equal. Several studies reported that the sink activity levels of cucumber and tomato vary with developmental stage and sink fruit position (Schapendonk and Brouwer, 1984; Marcelis, 1993; Wang et al., 1993). The present study showed that the same holds true for strawberry fruit. Similar tendencies in sink activity at the different fruit positions were obtained by integrative analysis of photosynthate translocation via ^13^C tracer measurement as shown in Figure 5B. However, the difference in sink activity level between the secondary and primary fruit determined by ^11^C (about 8 times) did not match that measured by ^13^C (about 3 times). Translocation data obtained by the ^13^C-tracer method reflects the total process including translocation to the sink fruit, retranslocation to the other organs, and respiratory photosynthate consumption. In contrast, the ^11^C-translocation data obtained by PETIS reflects real-time phloem transport to the sink fruit. The inequalities in fruit sink activity levels determined by ^11^C- and ^13^C-methods might be explained by the differences in the range of translocation process measured by each method. Each technique uses a different translocation measurement time scale. Therefore, ^11^C-translocation analysis with PETIS may be more effective tool than the ^13^C-tracer in evaluating true sink organ activity because the former excludes post-translocational processes. The architecture of a strawberry inflorescence is cymose (Jahn and Dana, 1970). Two lateral branches (secondary fruit) emerge from the main branch (primary fruit) and two tertiary branches (tertiary fruit) emerge from each secondary branch. This architecture causes flowering and fruit growth to proceed sequentially from the lower to the higher positions. As a result, the tertiary fruits at the higher position are smaller than the primary fruit at the lower position on the same inflorescence (Forney and Breen, 1985c). Bangerth (1989) suggested that auxin signaling may regulate the partitioning of photosynthates between competing sink fruits. According to this hypothesis, polar auxin export from the fruit that developed earlier (relatively high sink strength) inhibits auxin export from the fruit that formed later (relatively low sink strength) on the same inflorescence. This mechanism leads to the partitioning of more photosynthate to the sink fruit that developed earlier than to the sink fruit that formed later. It was reported that auxin may play a key role in strawberry fruit growth (Nitsch, 1950). Therefore, the auxin signal may induce sequential strawberry fruit growth in cymose inflorescence by allocating unequal amounts of photosynthate between the sink fruit developed earlier and those developed later. In this study, differences in the photosynthate translocation patterns from a specific leaf to the fruit at various positions might be the result of inequalities in the auxin signals from fruits with different sink activity. Forney and Breen (1985b) reported that unlike fruiting plants, deblossomed strawberry plants promoted dry matter partitioning to other organs but not to the inflorescence. Shishido et al. (1993) reported that in tomato plants, removal of the strong sink organ rerouted the photosynthate to another young growing sink. Therefore, removal of fruit 2 (which had the highest sink activity according to the ^11^C-translocation analysis) might change the photosynthate distribution pattern, as well as the quantities of ^11^C-translocation, to sink fruits 1 and 4. The actual amount of photosynthate allocated to these fruits may, in fact, increase depending on their sink activity. Further study is necessary to determine the effects of fruit picking on photosynthate translocation.

**Table 1:** ^11^C-photosynthate translocation rates from source leaf to Ped (fruit 1), Ped (fruit 2), and Ped (fruit 4), and from Ped (br) to Ped (fruit 1), Ped (fruit 2), and Ped (fruit 4).

**Translocation pathway interval**	**Length**	**^**11**^C arrival time**	**Translocation rate**
	**(cm)**	**(min)**	**(cm min^**−1**^)**
Leaf–Ped (fruit 1)	34.8	49	0.71
Leaf–Ped (fruit 2)	37.6	50	0.75
Leaf–Ped (fruit 4)	37.2	105	0.35
Ped (br)–Ped (fruit 1)	1.8	5	0.36
Ped (br)–Ped (fruit 2)	4.6	6	0.77
Ped (br)–Ped (fruit 4)	4.2	61	0.07

Translocation of ^11^C-photosynthate to fruits 3, 5, and 6 was not confirmed. Photosynthate translocation to sink organs may be associated with the phyllotaxis and arrangement of vascular system between each source and sink organ in tomato and potato plants (Shishido et al., 1988; Osaki et al., 1991; Shishido, 1991). In this study, we fed ^11^CO_2_ gas only to the fourth leaf immediately below the inflorescence. Unobserved ^11^C-translocation to the fruits may have been caused by the paucity of phloem connections between the source leaf and fruits 3, 5, and 6 relative to those between the source leaf and fruits 1, 2, and 4. Therefore, it might be suggested that photosynthate translocation from source leaf to sink fruit is closely associated with sink activity and the vascular system configuration between source leaves and sink fruits. Furthermore, a ^14^CO_2_ strawberry leaf feeding analysis revealed photosynthate translocation across multiple fruiting inflorescences (Kumakura and Shishido, 1994). Photosynthate translocation from a specific leaf to multiple inflorescence might be determined by the sink activity and the configuration of the vascular system between the source leaf and sink fruits on each inflorescence. However, this mechanism requires further investigation. In the present study, a specific leaf directly under an inflorescence was fed ^11^C- and ^13^C-labeled CO_2_, and the differences in sink activity and quantity of translocated photosynthate were measured in the fruits at various positions on the same inflorescence. However, those fruits may have also received photosynthate from the leaves at other positions since, as revealed in the present study, a single source leaf can supply photosynthates to multiple fruits. Therefore, the translocational data obtained for a specific leaf might not correspond to those for multiple source leaves. It is well-known that photosynthate translocation is largely influenced by environmental factors. Lanoue et al. (2018) reported that illuminations of ^14^C-fed tomato leaves with wavelength-specific LEDs enhanced carbon export to a greater extent in the daytime than in the night-time, and this day-time carbon export increased under the specific wavelength illuminating only at the medium photosynthetic rate. According to Yamazaki et al. (2015), elevation of atmospheric CO_2_ concentration from 400 to 1,500 ppm increased the amount of ^11^C-translocated photosynthates to tomato fruits with increasing carbon fixation. A rapid translocation response (within minutes) to changes in sink temperature has been reported for barley seedlings (Minchin et al., 2002). Walker and Ho (1977) reported that compared with the control fruit at 25°C, the photosynthate translocation rate was reduced by cooling the fruit to 5°C and accelerated by warming the fruit to 35°C. Kitano et al. (1998) also reported that the temperature rise treatment around fruit enhanced tomato fruit growth rate and sugar translocation rate, and they suggested that these effects are the result of the increases in energy-dependent translocational process through promoting the fruit respiration which occur as fruit temperature rises. Shishido et al. (1989) reported that in tomato plants, increases in nighttime temperatures accelerated photosynthate translocation, and changed distribution pattern of photosynthates; distribution to the lower parts of plant body was reduced while that to the upper parts was enhanced. Therefore, environmental control based on translocational responses could improve photosynthate translocation to strawberry fruits. In particular, temperature regulation near sink fruits may influence translocation rates and could be used to manipulate photosynthate distribution patterns in competing sinks. Further study is necessary to elucidate the effect of environmental factors on photosynthate translocation in strawberry plants. In the present study, insufficient data was gathered to verify the effect of vascular arrangement between multiple source leaves and sink fruits on translocation to the fruits. The ^11^C-translocation analysis by PETIS visualizes spatiotemporal-continuous connection of the photosynthate transport pathway. This non-invasive analysis enables the same plant to be used repeatedly in translocational experiments. Photosynthate translocation from source leaves at different positions to the same fruit sinks can be analyzed within the same plant. In contrast, this analysis cannot be performed using the conventional destructive ^13^C-labeling method. Therefore, sequential PETIS analyses of ^11^C-translocation from different source leaf positions to sink fruits may explain the influence of vascular arrangement on translocation between multiple source leaves and sink fruits. It could also elucidate translocation pathway structure between source leaves and sink fruits. This repeating PETIS analysis may also account for the effect of environmental factors on photosynthates translocation in strawberry plants. Further study using PETIS analysis is needed to determine the effects of leaf position and cultivation environments on photosynthate translocation to sink fruits, clarify the sink-source relationship, and identify the real-time translocation response to environmental conditions. Once it is applied, this information could enhance fruit yield and quality by improving the sink-source balance (numbers of leaves and fruits) and optimizing environmental conditions to suit protected strawberry cultivation.

## Conclusion

Non-invasive, real-time ^11^C photosynthate translocation analysis with PETIS successfully visualized the dynamics of photosynthate translocation from the individual source leaf immediately below the inflorescence to sink fruit in intact strawberry plant. This continuous spatiotemporal analysis elucidated photosynthate arrival times, revealed heterogeneous photosynthate accumulation within a fruit, and demonstrated that photosynthate accumulation from a specific leaf differs among fruit on various positions on the same inflorescence. These differences in photosynthate translocation among the fruits might be caused by variation in their relative sink activity levels. This study is the first to report real-time translocation to individual fruits on an inflorescence with physiological translocation parameters using ^11^C-radioactive- and ^13^C-stable-isotope analyses in strawberry plants.

## Author Contributions

KH, YM, and NK conceived and designed the experiments. KH, YM, SI, NS, Y-GY, KK, and NK performed the experiments. KH, YM, KN, and TA analyzed the data. KH and YM wrote the manuscript. All authors finalized the manuscript.

### Conflict of Interest Statement

The authors declare that the research was conducted in the absence of any commercial or financial relationships that could be construed as a potential conflict of interest.
